# The potential protective effect of Empagliflozin against Cisplatin-induced ovarian damage via upregulation of SIRT1/Nrf2 and amelioration ER Stress

**DOI:** 10.1007/s10787-025-01970-0

**Published:** 2025-09-30

**Authors:** Magda E. El-Sayad, Sally E. Abu-Risha, Sarah S. El-sisi, Hanaa A. Ibrahim

**Affiliations:** Department of Pharmacology and Toxicology, Faculty of Pharmacy, Tanta University, Tanta, 31527 Egypt

**Keywords:** Cisplatin, Empagliflozin, SIRT-1, NRF2, ER stress

## Abstract

Cisplatin (Cis) is a chemotherapeutic agent used for several types of malignant tumors. Though, its use causes several adverse effects as ovarian toxicity through its effect on unbalancing antioxidant and oxidants causing oxidative stress and inflammation. The present study aimed to examine the potential protective effect of Empagliflozin (EMPA) on Cisplatin-induced ovarian damage. Forty-four adult female albino rats were divided into four groups: control group, EMPA group (EMPA 10 mg/kg/day, P.O) for 17 days, CIS group (CIS 7 mg/kg, I.P) on day 14, Cis + EMPA group, EMPA (10 mg/kg/day, P.O) for 17 days with CIS (7 mg/kg) on day 14. Oxidative stress markers, inflammatory markers and endoplasmic reticulum stress markers of ovarian tissues, and ovarian SIRTuin-1 (SIRT-1) were analyzed. Histopathological examination of ovaries and capase-9 immunohistochemical staining were also evaluated. CIS significantly increased ovarian oxidative stress markers, ER stress markers and reduced both AMH levels and SIRT-1 expression. Histopathological findings showed ovarian toxicity, necrosis and positive capase-9 immuno-expression. EMPA significantly decreased both oxidative stress and ER stress biomarkers with a significant improvement in the histopathological findings and a decrease in caspase-9 immuno-expression.

Accordingly, EMPA protected against CIS-induced ovarian damage by activating both the SIRT-1/NRF2 /caspase-9 pathway and reducing ER stress.

## Introduction

Cancer remains one of the top reasons of death globally. Recent studies show that, in 2024 around 611.720 deaths because of cancer have occurred and 2,001,140 new cases are discovered in the US (Siegel et al. 2024). Although cancer treatment by cytotoxic chemotherapeutic agents has been improved and the mortality rate has been declining, chemotherapeutic agents can induce a variety of complications (Siegel et al. 2024; Cho et al. 2020).

Fertility problems have been documented in women receiving cytotoxic chemotherapeutic agents for decades (Cho et al. 2020). These agents can affect ovaries directly leading to follicular maturation dysfunction. A permanent decrease in several primordial follicles that cannot be replaced in adulthood, and a decrease in anti-Mullerian hormone (AMH) and change in ovarian histopathology with the presence of fibrosis and ischemia have been documented (Algandaby 2021; E. kaygusuzoglu et al. 2018; Sancho-Martínez et al. 2012).

Cisplatin (cis-diamminedichloroplatinum II) is an alkylating agent and a platinum derivative that has been used as a chemotherapeutic agent to treat various types of solid tumors, including colon, lung, ovarian, testicular, cervical, and breast cancer (Ibrahim et al. 2021; Dasari and Bernard Tchounwou 2014). CIS cytotoxicity has not been specific for tumor cells, but it also affects normal cells causing a disturbance in the physiological functions of different body systems (Sancho-Martínez et al. 2012).

One of the most common adverse effects of CIS is ovarian toxicity, leading to the depletion of primordial follicles (E. kaygusuzoglu et al. 2018). The exact mechanism of CIS-induced-ovarian toxicity is not yet fully understood (E. kaygusuzoglu et al. 2018). Multiple mechanisms have been proposed, evidence suggests that oxidative stress plays an important role in CIS-induced ovarian toxicity leading to apoptosis and eventually necrosis (Nguyen et al. 2019; Ayazoglu Demir et al. 2022; Moslehi et al. 2023). Recent studies shed light on the role of endoplasmic reticulum (ER) stress in CIS cytotoxicity. However, the exact mechanism by which CIS activates ER stress leading to the activation of caspases and apoptosis is still not known (Demir 2024; Demir et al. 2023).

Sodium-glucose cotransporter-2 (SGLT-2) inhibitors are hypoglycemic agents that inhibit renal glucose reabsorption, thereby increasing urinary glucose excretion. SGLT-2 inhibitors have been widely used recently in different conditions both as potentially protective or as a treatment (Kontana and Tziomalos 2019). SGLT-2 inhibitors have shown to possess a powerful anti-oxidant properties. Suggested evidence shows that SGLT-2 inhibitors can exert pleiotropic effects via various mechanisms of action, they decrease oxidative stress, apoptosis of cells and activate autophagy through various pathways (Tsai et al. 2021).

They reduce free radical and increase antioxidants such as glutathione (GSH), superoxide dismutase (SOD), and decrease inflammation and expression of various inflammatory markers such as tumor necrosis factor (TNF-α), interleukin 6 (IL-6), nuclear factor-κB (NF-κB), and C-reactive protein (CRP) (Nabrdalik-Leśniak et al. 2023).

It has also been proposed that empagliflozin (EMPA), SGLT-2 inhibitor, exerts its anti-oxidant activity through activation of the SIRT1/NRF2 pathway, regulating transcription of several anti-oxidant proteins, eventually attenuating cell apoptosis (Abdelzaher et al. 2023).

The ER stress is another possible mechanism by which EMPA attenuates oxidative stress and cell apoptosis, modulating and improving misfolded protein clearance (Nasiri-Ansari et al. 2021; Androutsakos et al. 2022; Lee et al. 2024).

In this study, we aim to investigate the potential protective role of EMPA in CIS-induced ovarian damage, and examine the mechanisms involved in such potential effect.

## Materials and methods

### Chemicals and drugs

CIS vials for injection (Cisplatine)® (50 mg/50 mL) was purchased from viatris. EMPA powder was obtained from Sigma pharmaceuticals (Cairo, Egypt).

### Animals and study design

Forty-four adult female albino rats, weighing about 180–220 g, were purchased from the national research center’s animal house (Giza, Egypt). Rats were kept in the animal house at the Faculty of Pharmacy, Tanta University, Tanta, Egypt. Rats were housed in a standard housing (5 rats per cage) on a 12 h light–dark cycle at 25 ± 2 °C, and had 24 h accesses to tap water and chow. and left seven days before the start of the experiment for accommodation. The study was approved by the Research Ethics Committee (Faculty of Pharmacy, Tanta University, Egypt) and complied with the guidelines of The Council for International Organizations of Medical Sciences (CIOMS) (TP/ RE/1/24M-004).

Rats were randomly divided into four groups; each group have 11 rats.

1. *Control group*: Rats received normal saline for 17 days.
2. *EMPA group*: Rats received EMPA (10 mg/kg, P.O.) for 17 days.
3. *CIS group*: Rats received saline for 17 days and CIS (7 mg/kg, I.P.) was injected on day 14.
4. *EMPA* + *CIS*: Rats received EMPA (10 mg/kg, P.O.) for 17 days and CIS (7 mg/kg, I.P.) was injected at day 14.

### Blood and tissue samples

At day 17, rats were anesthetized. Blood samples were then collected via cardiac puncture, centrifuged for 15 min and serum was taken and kept stored at – 20 °C for later use. Tissue samples of ovaries were then collected, washed with saline and divided into two parts, first part was stored in − 80 °C and second part was fixed in 10% buffered formalin for histopathological and immunohistochemical assessment.

### Biochemical analysis

Malondialdehyde (MDA) (MyBioSource, California) (Cat. No: MBS268427), superoxide dismutase (SOD)(Cusabio, Texas, USA) (Cat. No.: CSB-E08555r), anti-Mullerian hormone (AMH) (Assay Genie, Dublin, Ireland) (Cat. No.: RTFI01398), The nuclear factor erythroid 2– related factor 2 (Nrf2) (Bioassay technology laboratory, Shanghai, China) (Cat. No.: E1083Ra) and Glucose-Regulated Protein 78 (GRP78) (San Diego, USA) (Cat. No.: MBS2533524) were estimated by rat enzyme-linked immunosorbent assay (ELISA) kits, following manufacturer’s instructions.

### Histopathological analysis

Ovaries were obtained, and fixed in 10% buffered formalin for 48 h, then washed and dehydrated by ethanol, then following standard procedure, tissue sections were obtained and stained with hematoxylin and eosin (H&E)) and examined by a light microscope. The ovarian histopathological damage score was evaluated based on the following parameters: follicle cell degeneration, vascular congestion, hemorrhage, and inflammation (0: none, 1: mild, 2: moderate, 3: severe**)** (Karakaş et al. 2020).

### Immunohistochemical analysis

Tissue expression of caspase-9 was done by immunohistochemical analysis following standard procedures, with counterstain Haematoxylin was used. The mean area percentage of capsase-9 immunostaining in 5 high power fields at 400 magnification of tissue was performed using image j analysis software.

### Real-time polymerase chain reaction (RT-qPCR) analysis of SIRT1

Ovarian tissues were homogenized and RNA was extracted via a commercial kit (RNeasy Mini Kit Catalogue no.74104). Reverse transcription was done by cDNA reverse transcription kit (RevertAid Reverse Transcriptase (Thermo Fisher) (200 U/µL). Catalog number: EP0441). The RNA quantity and quality were subsequently assessed by measuring the absorbance ratio (A260/A280) using a Beckman dual spectrophotometer. Silent information regulator transcript-1 (SIRT1) expression was determined and the relative gene expression was calculated by the 2-ΔΔ method and normalized to Ct β-actin (Luo, et al. 2017) (Table 1).

$${\text{Whereas }}\Delta \Delta Ct \, = \, \Delta Ct{\text{ reference }}{-} \, \Delta Ct{\text{ target}}$$

$$\Delta Ct{\text{ target}} = Ct{\text{ control}}{-}Ct{\text{ treatment}}\,{\text{and }}\Delta Ct{\text{ reference}} = Ct{\text{ control}} - Ct{\text{ treatment}}$$

**Table 1:** Primer sequences of the tested genes

Gene	Primer sequence (5′-3′)	References
Mice ß-actin (housekeeping)	GTG GGA ATT CGT CAG AAG GAC TCC TAT GTG	Sisto et al. 2003
	GAA GTC TAG AGC AAC ATA GCA CAG CTT CTC	
Mice Sirt1	CGGCTACCGAGGTCCATATAC	Luo et al. 2017
	CAGCTCAGGTGGAGGAATTGT	

### Statistical analysis

For parametric data, one-way ANOVA followed by Tukey’s test were used. Significance was set at p value < 0.05. Results are shown as mean ± SD. While non-parametric data were analyzed by Kruskal Wallis test followed by Dunn’s post hoc test for Pairwise Comparisons. Values of histopathological scoring, expressed as median (minimum–maximum), A significant difference of < 0.05 was accepted. GraphPad Prism 9.5 Demo (GraphPad Software, San Diego, CA) was used for statistical analysis.

## Results

### Effect of EMPA on AMH content

**Fig. 1:**
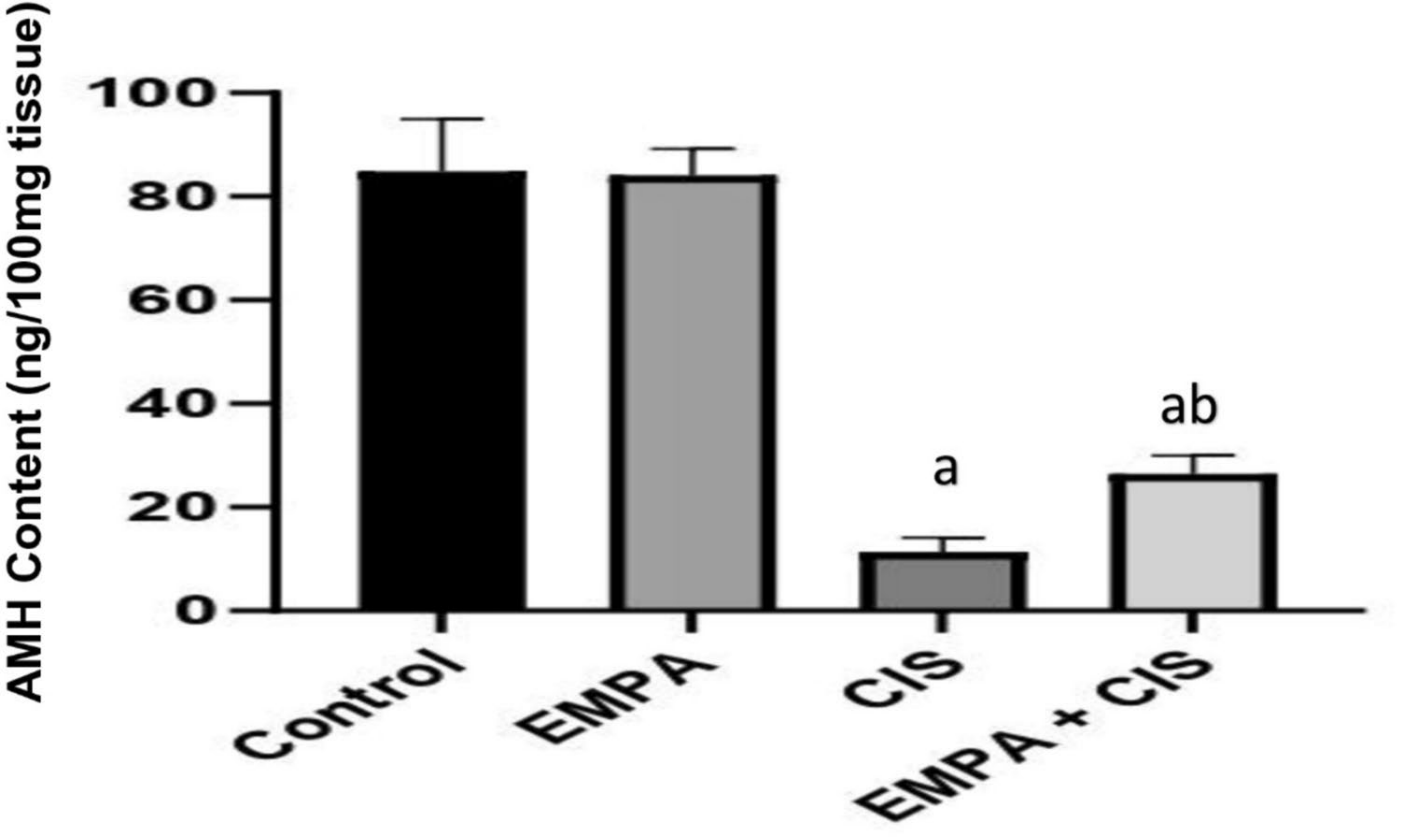
Effects of EMPA on AMH content. Data are expressed as mean ± SD, P < 0.05, (n = 5/group). CIS, CIS; and EMPA, EMPA. ^a^significance from normal control, ^b^significance from CIS

CIS treated group showed significant reduction in AMH content by 86% compared to control group, while Co-administration of EMPA with CIS showed significant increase in AMH content by 130% compared to CIS treated group alone (Fig. 1).

### Effect of EMPA on histopathological examination

**Fig. 2:**
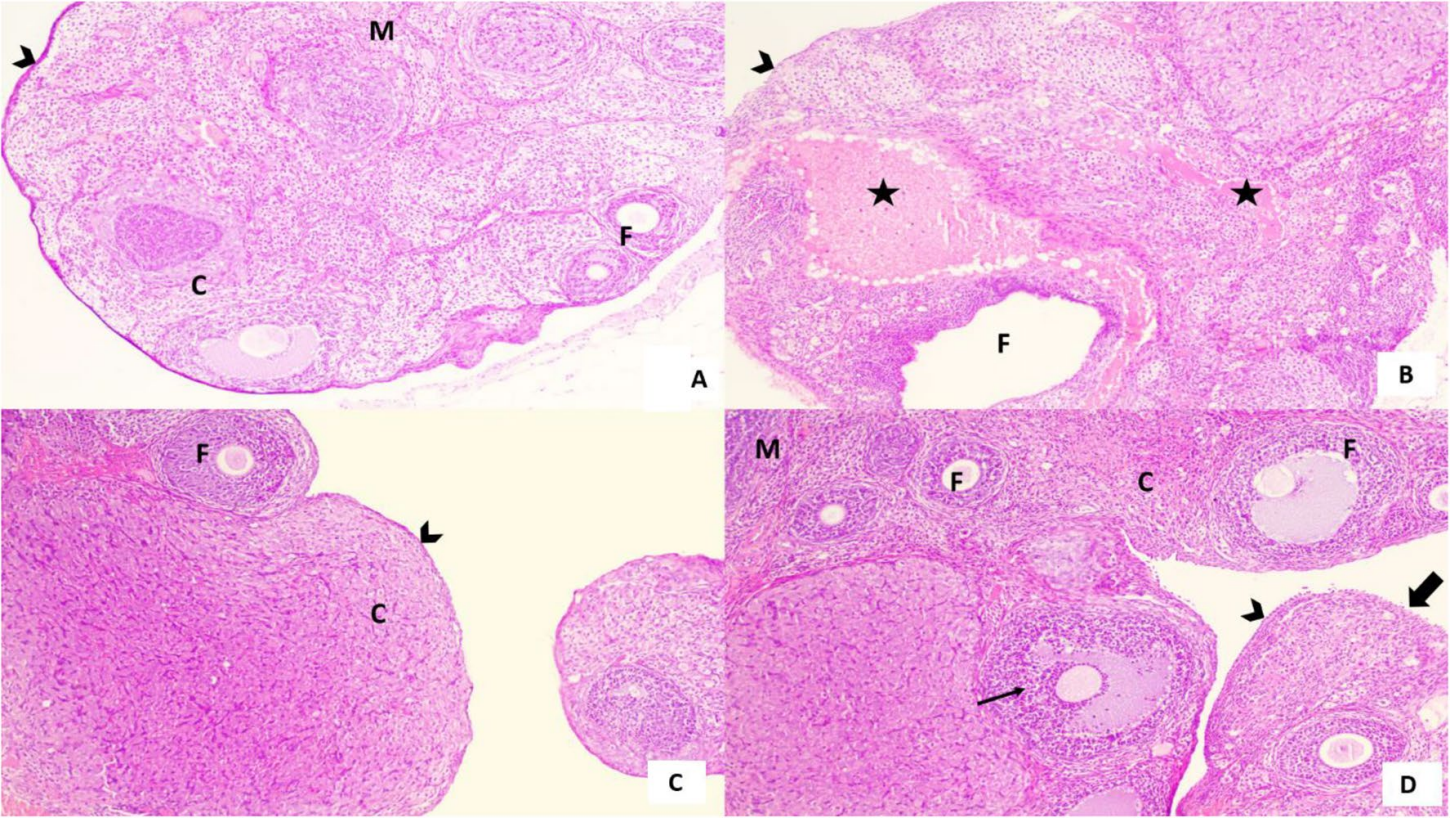
Effect of EMPA on histopathological examination of ovarian sections. Figure 3A. A section of the ovary of control group showing normal histological architecture consisting of outer cortex (**C**) covered by capsule with germinal epithelium (arrowhead) and inner medulla (**M**). The cortex containing multiple ovarian follicles (**F**). **B** A section of the ovary of EMPA group showing normal histological architecture similar to control group consisting of outer cortex (**C**) covered by capsule with germinal epithelium (arrow head) containing ovarian follicle (**F**). **C** A section of the ovary of CIS treated group showing disturbed histological architecture irregular capsule (arrow head), dilated ovarian follicle (**F**) and marked interstitial hemorrhage (*). **D** A section of the ovary of EMPA + CIS treated group showing marked improvement of histological architecture more or less similar to control group consisting of outer cortex (**C**) covered by intact capsule with germinal epithelium (arrow head) in most areas except for focal loss of capsule and germinal epithelium (thick arrow). The cortex contains multiple ovarian follicles (**F**) with disturbed arrangement of granulosa cells in one of them (thin arrow) and inner medulla was seen (**M**). **A**–**D** scale bar = 200 μm

An ovarian tissue section of EMPA treated group showing normal histological structure consisting of ovarian follicles, stroma, primordial follicle and normal vascular structure consistent with histological structure of control group. While CIS treated group showed dilated and congested blood vessels along with degenerated necrotic cells, interstitial hemorrhage throughout the tissue and absence of normal cell structure. Co-administration of EMPA with CIS showed marked improvement in histological structure similar to that of control and EMPA treated group, mild dilated blood vessel, less necrotic and degenerated cells and presence of normal cell structure with mature follicle containing oocyte (Fig. 2, Table 2).

**Table 2:** Histopathological analysis of Ovarian tissues

	Control	EMPA	CIS	EMPA + CIS
Disrupted histological architecture	0.0 [0.0—0.0]	0.0 [0.0—0.0]	3.0^**a**^ [3.0—3.0]	0.0^**b**^ [0.0—0.0]
Degenerated ovarian follicles	0.0 [0.0—0.0]	0.0 [0.0—0.0]	3.0^**a**^ [3.0—3.0]	1.0^**b**^ [0.0—1.0]
Hemorrhage	0.0 [0.0—0.0]	0.0 [0.0—0.0]	3.0^**a**^ [3.0—3.0]	0.0^**b**^ [0.0—0.0]
Dilated congested blood vessels	0.0 [0.0—0.0]	0.0 [0.0—0.0]	3.0^**a**^ [3.0—3.0]	1.0^**b**^ [0.0—1.0]

Data were represented as median plus [interquantile range]. Data were analysed by Kruskal–Wallis test, followed by Dunn’s multiple comparisons test., P < 0.05, (n = 5/group). CIS, CIS; and EMPA, EMPA

^a^significance from normal control

^b^significance from CIS

### Effect of EMPA on immunohistochemistry of caspase-9

**Fig. 3:**
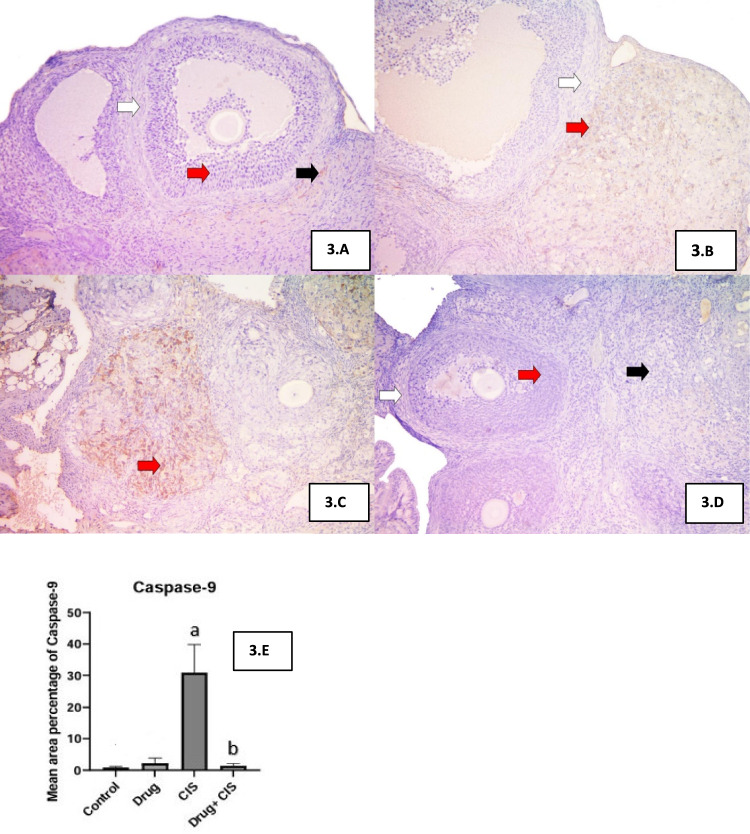
Effect of EMPA on immunohistochemistry of caspase-9. **A** Section of the ovary of control group I showing negative reaction in granulosa cells (red arrow), theca cells (white arrow) and weak reaction in stromal cells (black arrow). **B** A section of the ovary of EMPA group III showing weak reaction in granulosa lutein cells (red arrow) while negative reaction in theca cells (white arrow). **C** A section of the ovary of CIS treated group II showing strong positive reaction in granulosa lutein cells (red arrow). **D** A section of the ovary EMPA + CIS treated group IV showing negative reaction in granulosa cells (red arrow), theca cells (white arrow) and stromal cells (black arrow) similar to control group. **A**–**D** scale bar = 200 µm. **E** Effects of EMPA on caspase 9 mean are percentage. Data are expressed as mean ± SD, P < 0.05, (n = 5/group). CIS, CIS; and EMPA, EMPA. ^a^significance from normal control, ^b^significance from CIS

Control group and EMPA treated group showed negative to weak immunoreaction for caspase-9 in granulosa cells, while in CIS group showed a strong immunoreaction in granulosa cells, co-administration of EMPA with CIS lead to a negative reaction similar to the control group as well (Fig. 3).

As shown in Fig. 3E, treatment with CIS showed 3534% increase in area compared to control group, while co-administration with EMPA showed 2122% decrease compared to CIS treated group.

### Effect of EMPA on MDA content

**Fig. 4:**
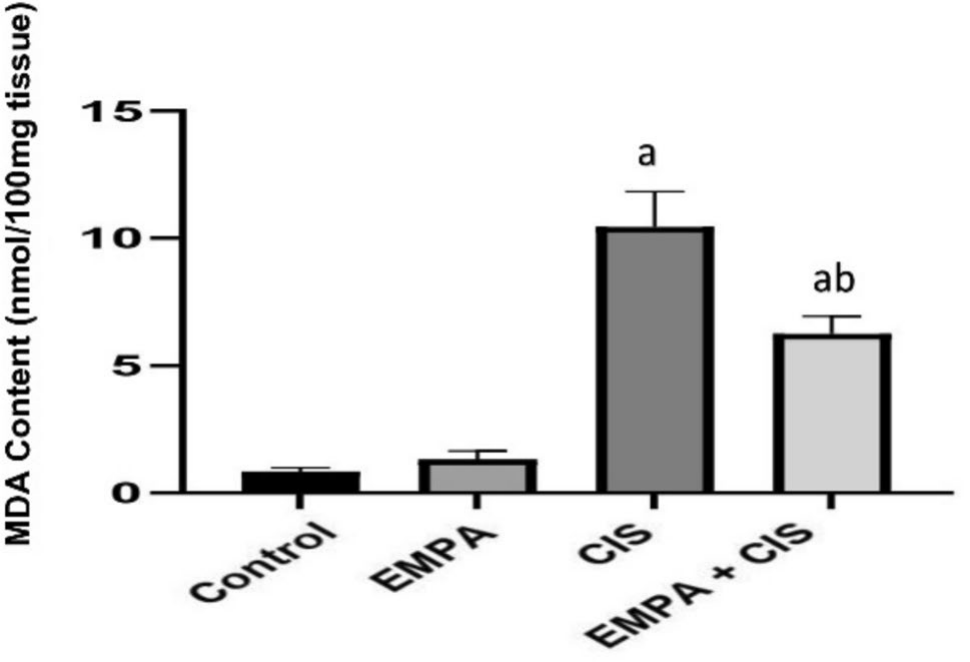
Effects of EMPA on MDA content. Data are expressed as mean ± SD, P < 0.05, (n = 5/group). CIS, CIS; and EMPA, EMPA. ^a^significance from normal control, ^b^significance from CIS

As shown in Fig. 4. CIS treated group showed significant increase in MDA content by 40% compared to control group, while Co-administration of EMPA with CIS showed significantly decreased in MDA content by 1127% compared to CIS treated group.

### Effect of EMPA on SOD content

**Fig. 5:**
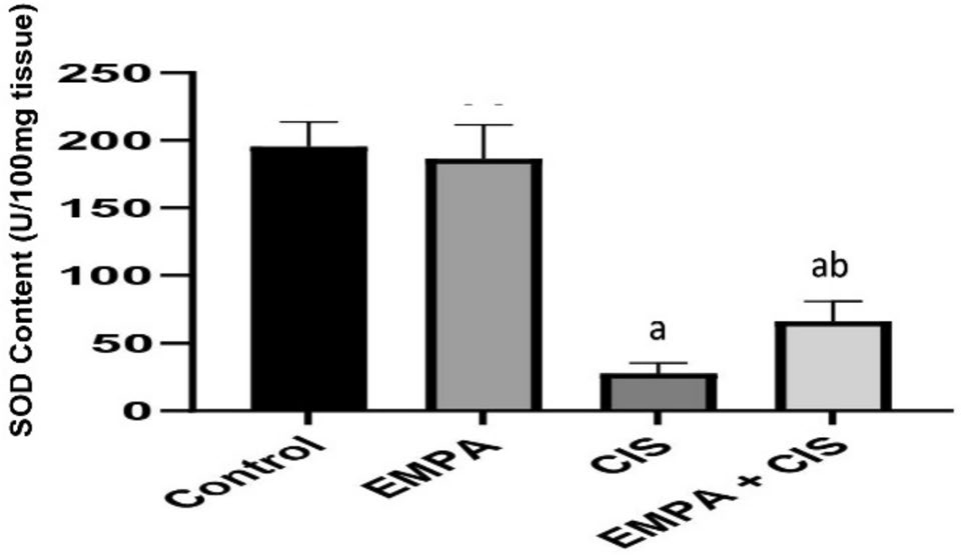
Effects of EMPA on SOD content. Data are expressed as mean ± SD, P < 0.05, (n = 5/group). CIS, CIS; and EMPA, EMPA. ^a^significance from normal control, ^b^significance from CIS

As shown in Fig. 5. CIS treated group showed significantly decreased in SOD by 85% compared to control, while co-administration of EMPA significantly increased SOD by 130% compared to CIS treated group.

### Effect of EMPA on NRF2 content

**Fig. 6:**
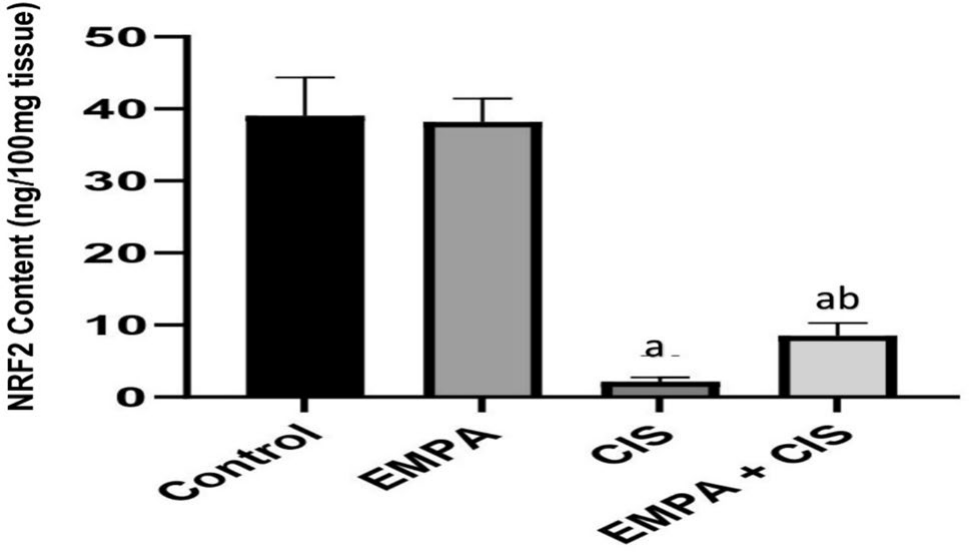
Effects of EMPA on NRF2 content. Data are expressed as mean ± SD, P < 0.05, (n = 5/group). CIS, CIS; and EMPA, EMPA. ^a^significance from normal control, ^b^significance from CIS

In comparison to control and Control group, CIS significantly decreased NRF2 by 94%. While treatment with EMPA to CIS, showed significant increase of NRF2 by 299% compared to CIS treated group alone (Fig. 6).

### Effect of EMPA on ER stress

**Fig. 7:**
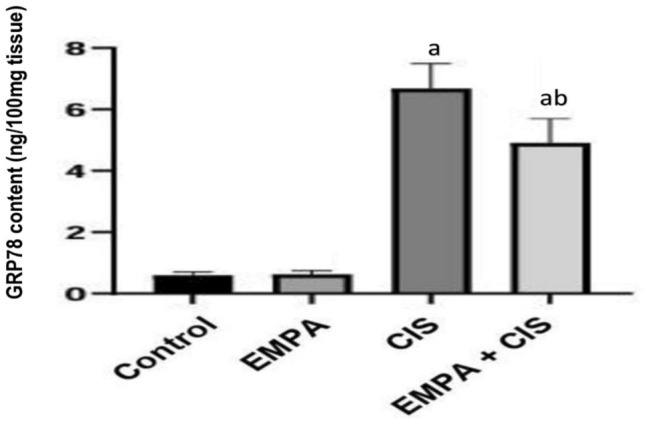
Effects of EMPA on GRP78 content. Data are expressed as mean ± SD, P < 0.05, (n = 5/group). CIS, CIS; and EMPA, EMPA. ^a^significance from normal control, ^b^significance from CIS

The ER stress was assessed by analysis of GRP78, Control group showed significantly decreased level of GRP78 by 1012% compared to CIS treated group, while administration of EMPA with CIS showed significant reduction of GRP78 by 26.4% compared to CIS treated group (Fig. 7).

### Effect of EMPA on SIRT1 expression

**Fig. 8:**
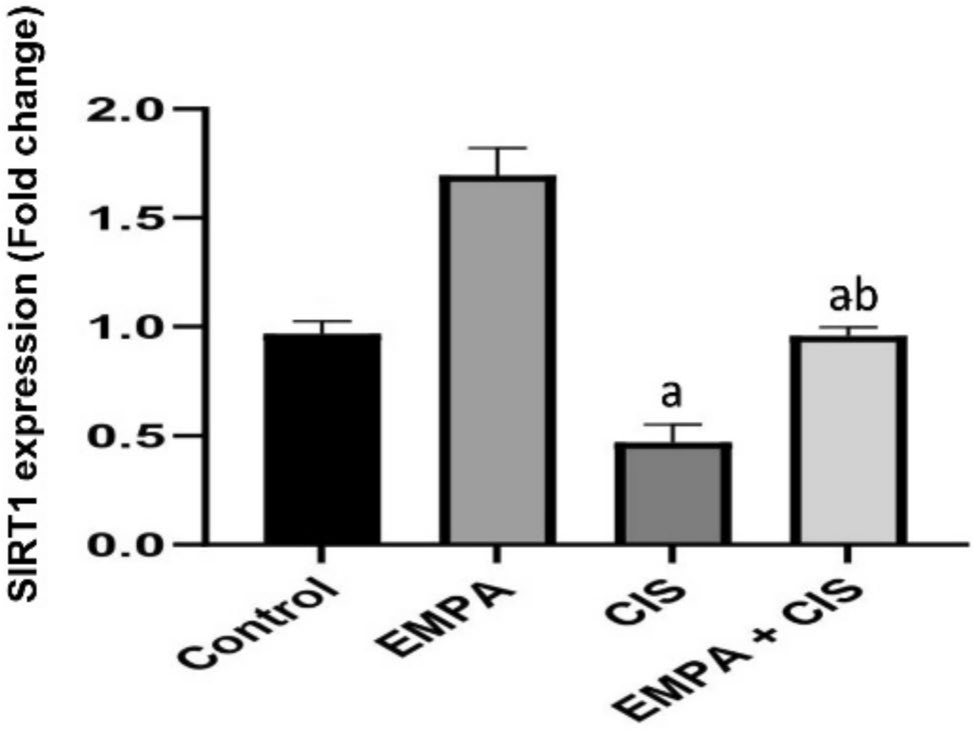
Effects of EMPA on SIRT1 content. Data are expressed as mean ± SD, P < 0.05, (n = 5/group). CIS, CIS; and EMPA, EMPA. ^a^significance from normal control, ^b^significance from CIS

As shown in Fig. 8. CIS treated group showed significant decrease in SIRT1 ovarian expression by 52% compared to control group, while EMPA + CIS treated group showed significant increase in ovarian expression of SIRT1 by 102% compared to CIS treated group.

## Discussion

Cisplatin has been widely used as a chemotherapeutic agent for many solid tumors, however numerous side effects are caused by CIS (Debersac et al. 2001). Studies have shown that female patients treated with CIS for malignancy, have a higher risk of fertility problems and extensive ovarian damage with primordial follicular depletion (Al-Shahat et al. 2022; Debersac et al. 2001).

Most studies have shown role of oxidative stress as a key mechanism for CIS-induced ovarian toxicity (Al-Shahat et al. 2022; Altuner et al. 2013). Hence, In the current study, we have examined the possible mechanisms by which EMPA could attenuate CIS-induced ovarian damage.

The ovarian toxicity of CIS was examined by its effect on AMH production, this result was further confirmed by histopathological examination, this result was further confirmed by histopathological examination that shows marked interstitial hemorrhage, degenerated ovarian follicles and disrupted histological architecture.

The AMH is constantly used as a marker of follicles’ reservoir inside the ovary and how much follicles left (Tas et al. 2019), It’s an earlier predictor of ovaries reserve than other ovarian hormones as FSH as shown in previous studies (Yeh et al. 2009).

Early studies showed negative effect of CIS on AMH level indicating decreased primordial follicles due to follicular apoptosis by CIS (Anğın et al. 2020), as well as decrease in primary and secondary follicles in patients receiving CIS compared to control groups (Tutar et al. 2022).

Several studies showed as well decreased level of fertility hormones with CIS treatment, decreased AMH level indicates poor fertility, ovarian and follicles damage. Yeh et al. (2009) and may reflect a feedback mechanism from ovaries due to damage and depletion of primordial follicles (Iwase et al. 2023), which was previously shown in histopathological results.

Notably, pretreatment with EMPA showed an increase in AMH production and improved histopathological changes, indicating that EMPA can protect against CIS-induced ovarian damage. Abdelzaher et al*.* showed the protective effect of EMPA against haloperidol-induced ovarian damage (16), To our knowledge, this is the first study investigating the potential protective effect of EMPA in CIS-induced ovarian toxicity.

Oxidative stress has emerged as a key factor mechanism in CIS-induced ovarian toxicity and failure, affecting AMH production and causing follicles depletion. The present study showed similar evidence, that CIS caused ovarian damage through an imbalance between oxidants and antioxidants. CIS showed marked decrease in SOD, an antioxidant enzyme, and marked elevation in MDA compared to control group.

Malondialdehyde has been widely used as a marker for lipid peroxidation and oxidative stress as well, treatment with CIS in this study and in previous studies as well showed high level of MDA content due to effect of CIS on lipid peroxidation in ovarian tissue (Maurya et al. 2021; Li et al. 2013), this due to imbalance between oxidants and antioxidants during treatment with CIS.

Recent studies reveal the important role of activation the SIRT1/NRF2 pathway as a major modulator for anti-oxidant, anti-inflammatory and anti-apoptotic pathways (Ren et al. 2019; Abu-Baih et al. 2024). More specifically, increasing evidence showed that SIRT1 may upregulate NRF2 which is a transcription factor known to play a prominent role in modulating many downstream proteins that regulate oxidative stress and up regulate anti-oxidants activity such as SOD (Tang et al. 2024). During oxidative stress, NRF2 become consumed causing disruption in oxidant and anti-oxidant homeostasis (Liu et al. 2021). This aligns with our present study that showed that CIS markedly decreased SIRT1 expression with subsequent decrease in NRF2 and eventually decrease antioxidants which explains low level of SOD and increase in MDA compared to control group. In the present study, EMPA reversed the consumption of SIRT1 and NRF2 caused by CIS exposure.

Relationship between EMPA and SIRT1 has been discussed recently, it has been proposed that EMPA may activate SIRT1 by upregulation of SIRT1 mRNA as was suggested by Tian et al. (2021) which explains the powerful antioxidants effect of SGLT2 inhibitors in various models; cardiology and nephrology, and could potentially prevent degradation of SIRT1 protein, although further studies are needed to confirm this.

It has been shown that oxidative stress result in activation of apoptotic pathway and activation of caspases (Abdelzaher et al. 2023). While increase in SIRT1 represses ovarian apoptosis through activation of anti-oxidants. This was proven by our current study which showed positive immunoreaction in caspase 9 in CIS treated group as compared to control group. Increase in caspase 9 usually lead to activation of caspase 3 which is the final step for initiation of apoptotic cascade and cell death (Dirican et al. 2023).

Interestingly in our study, EMPA has shown marked increase in SIRT1 activity even compared to control group. This explains the upregulation in NRF2; inhibiting oxidative stress with subsequent marked decrease in MDA level and increasing anti-oxidants activity with subsequent increase in SOD level, eventually attenuating the inflammatory process and preventing initiation of apoptotic pathway, which was proven by decreased level of caspase-9 shown by immunostaining of ovarian tissue.

Recent studies have investigated the potential association between SIRT1 inhibition and activation of ER stress through The UPR signaling pathway (Hu et al. 2024). Mechanistically, when SIRT1 expression decreases in the cell this led to dissociation of UPR transmembrane sensors from GRP78 protein, activating ER stress leading to protein degradation, causing cellular damage, activation of caspases and eventually apoptosis (Hu et al. 2024; Liu et al. 2023).

That align with our study, where as in CIS treated group high level of GRP78 was observed indicating activation of ER stress by CIS. Consequently, activation of oxidative stress and ER stress by CIS explains high level of caspase 9 that was observed causing ovarian failure, necrosis and hemorrhage.

EMPA, an SGLT2 inhibitor has been extensively studied at several organs as a powerful anti-oxidant, leading to protection of cells from degradation and apoptosis through different anti-inflammatory pathways (Macha et al. 2014; Li et al. 2021). EMPA may increase activity of SIRT1, subsequently leading to increase in NRF2 and antioxidants, protecting from extensive oxidative stress, inflammation and apoptosis (Abdelzaher et al. 2023).

Increased SIRT1 expression by EMPA although led to downregulation of GRP78 which decreased the dissociation of UPR transmembrane sensors indicating lower activity of ER stress, protecting cell from protein degradation and further cellular and protein damage.

**Fig.9:**
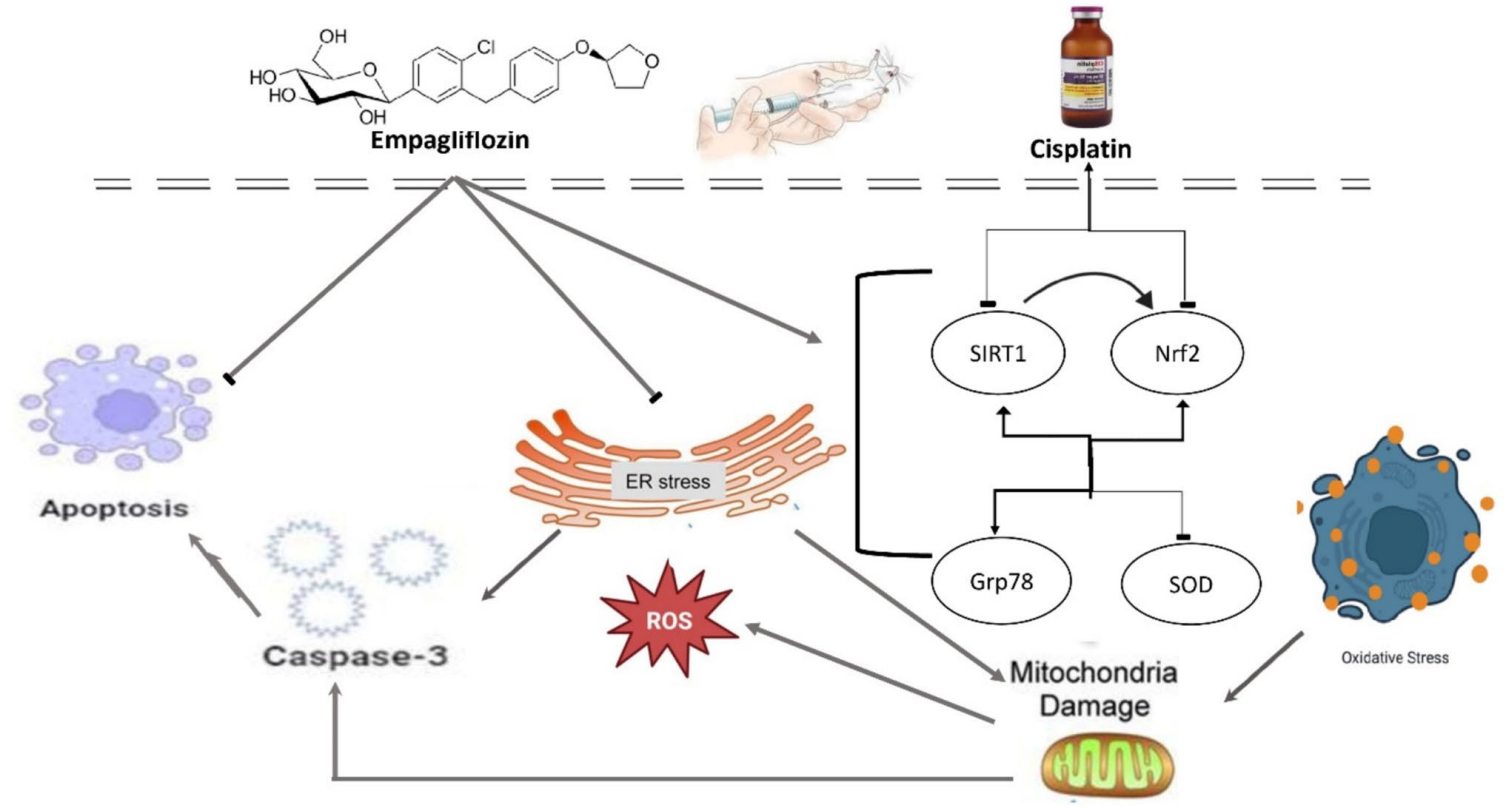
Graph summarizing the mechanisms of Cis-induced ovarian toxicity and the protective effect of Empagliflozin. *Sirt-1* Sirtuin-1, *Nrf2* nuclear factor erythroid 2-related factor 2, *Grp78* Glucose-Regulated Protein 78 (GRP78), *SOD* superoxide dismutase

Collectively, EMPA may works on several pathways; inhibition of oxidative stress and ER stress, leading to powerful protection against CIS-induced cellular damage and necrosis in ovaries. (Fig. 9) is a self abstracted diaghram to describe the mechanisms involved in the study.

## Conclusion

Our study showed that EMPA has a beneficial protective effect in CIS-induced ovarian damage, through various mechanisms, increasing antioxidants by activation of SIRT1/NRF2 pathway and decreasing ER stress by activation of SIRT1/GRP78 pathway. Both pathways caused decreased apoptosis, improved AMH level and ovarian follicles and oocytes.

## Data Availability

Data will be made available on request.
